# Increased risk of cerebrovascular accident related to non-alcoholic fatty liver disease: a meta-analysis

**DOI:** 10.18632/oncotarget.22755

**Published:** 2017-11-29

**Authors:** Jianping Hu, Yong Xu, Zemin He, Hui Zhang, Xiaoqing Lian, Tiantian Zhu, Caihong Liang, Jun Li

**Affiliations:** ^1^ Department of General Surgery, The Affiliated Jiangning Hospital of Nanjing Medical University, Nanjing, Jiangsu Province, P.R. China; ^2^ Department of Nephrology, Huai'an Second People's Hospital and The Affiliated Huai'an Hospital of Xuzhou Medical University, Huai'an, China; ^3^ Department of Cardiovasology, The Affiliated Jiangning Hospital of Nanjing Medical University, Nanjing, Jiangsu Province, P.R. China

**Keywords:** cerebrovascular accident, non-alcoholic fatty liver disease, meta-analysis

## Abstract

Recent published studies on the association between non-alcoholic fatty liver disease (NAFLD) and cerebrovascular accident (CVA) risk have yielded conflicting findings. The aim of our study was to identify the potential association by pooling all available publications. A total of nine independent studies were included into our study. The pooled odd ratio (OR) with 95% confidence interval (95% CI) was calculated to weigh the strength for the relationship between NAFLD and CVA risk. We also conducted stratified analyses by study design, ethnicity and disease classification for further elucidation. The pooled results of the present meta-analysis showed that NAFLD was related to increased risk of CVA (OR = 2.32, 95% CI 1.84–2.93, *P* < 0.001). Besides, NAFLD is associated with increased risk of CVA among both Caucasians (OR = 2.27, 95% CI 1.77–2.90, *P* < 0.001) and Asians (OR = 2.81, 95% CI 1.43–5.51, *P* = 0.003). Moreover, the significant association was also observed in case-control studies (OR = 2.73, 95% CI 1.67–4.48, *P* < 0.001) and cohort studies (OR = 2.22, 95% CI 1.71–2.89, *P* < 0.001), respectively. In addition, NAFLD was shown to correlate with increased risk of cerebral hemorrhage (OR = 1.85, 95% CI 1.05–3.27, *P* = 0.034) and the ischemic stroke (OR = 2.51, 95% CI 1.92–3.28, *P* < 0.001). In conclusion, our findings firstly provide strong evidence for a risk effect of NAFLD on CVA development.

## INTRODUCTION

Cerebrovascular accident (CVA), also known as stroke, mainly includes ischemic stroke due to lack of blood and hemorrhagic stroke due to bleeding in the brain. CVA is the second most common disease of death and the major cause of disability around the world and third to fourth leading cause of death and the main cause of disability in the United States [1, 2]. Stroke can affect survival, mentality, emotion, or a combination of the three, and the disability caused by a stroke makes survivors decrease their employability. CVA already becomes a global severe problem and leads to heavy economic burden worldwide. A number of studies have implicated that non-alcoholic fatty liver disease (NAFLD) plays an important role in the development of CVA [3–5].

NAFLD is characterized by the presence of significant lipid accumulation in the liver parenchyma without excessive alcohol ingestion and any other liver diseases. In recent years, NAFLD has already become a familiar clinical diagnosis and its prevalence and incidence increase dramatically and consistently in the general population [6]. Due to incorrect dietary habits and sedentary lifestyle, NAFLD is frequent in populations from United States, Europe, Middle-East, Australia, China and Japan and NAFLD is a common liver condition with a prevalence of 20–30% globally [7]. NAFLD can be categorized into nonalcoholic steatohepatitis (NASH) and nonalcoholic fatty liver (NAFL) by histology [8]. The clinical manifestations of patients with NAFLD may range from asymptomatic, transaminase rising to cirrhosis and hepatocellular carcinoma [9]. Some studies have investigated the association between NAFLD and CVA risk. Several studies showed that NAFLD played a risk role in CVA [10, 11], while another study indicated that NAFLD is not independently related to ischemic stroke [5]. Thus, previously available data estimating the role of NAFLD in CVA risk are inconsistent and inconclusive, probably due to a diverse population, race, study design, and CVA classification. As a result, we for the first time carry out a meta-analysis of all currently published studies to shed some light on the contradictory findings and provide a more precise estimate for the association between NAFLD and CVA risk.

## RESULTS

### Identification and characteristics of eligible studies

**Figure 1:**
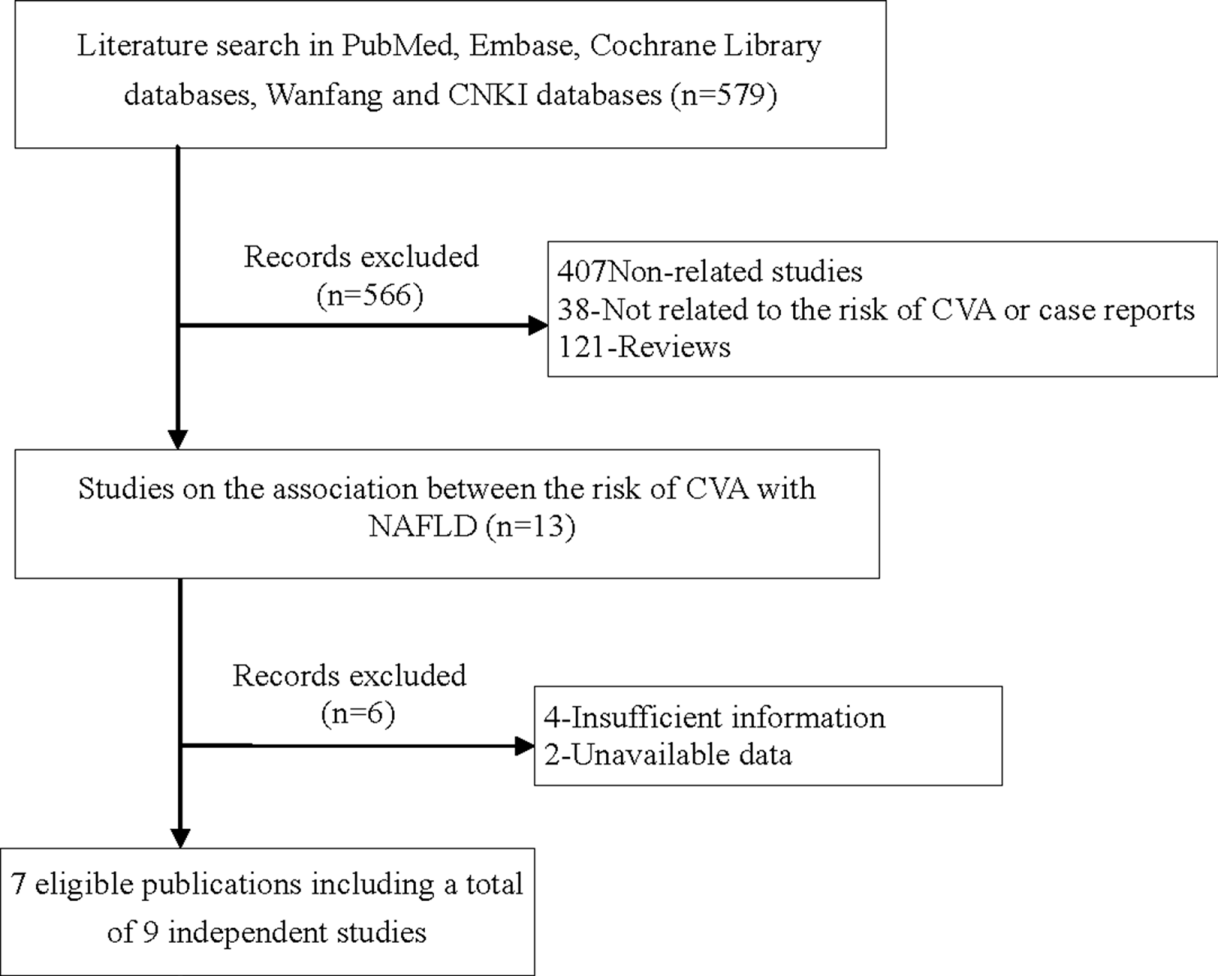
Flow chart for identification of eligible studies

After a comprehensive search in PubMed, Embase, Cochrane Library databases, Wanfang and CNKI databases up to Sep 30, 2017, a total of 7 publications were retrieved regarding the association between NAFLD and CVA [3–5, 10–12]. Among them, 2 publications were regarded as 4 independent studies according to different CVA [10, 11]. As a result, we included 9 individual studies. Figure 1 showed details for the inclusion of all eligible studies. Characteristics of all included studies were shown in Table 1. Among the 9 studies, 2 were in case-control design, while 7 were in cohort design. 6 independent studies were conducted among Caucasians, and 3 were among Asians.

**Table 1:** Descriptive characteristics of all studies

Study	Year	Location	Ethnicity	Study design	Diseases	CVA			Control			Quality assessment Score^*^
						Number	Age (year)	Gender (male/female)	Number	Age (year)	Gender (male/female)	
Fracanzani AL	2016	Italy	Caucasians	Cohort	Ischemic Stroke	6	NR	NR	267	NR	NR	7
Moshayedi H	2014	Iran	Caucasians	Case-Control	Ischemic Stroke	110	66.42 ± 11.31	69/41	110	66.51 ± 11.27	69/41	7
El Azeem HA (1)	2013	Egypt	Caucasians	Cohort	Ischemic Stroke	82	NR	NR	665	NR	NR	8
El Azeem HA (2)	2013	Egypt	Caucasians	Cohort	Cerebral Hemorrhage	49	NR	NR	698	NR	NR	8
Pickhardt PJ	2013	Korea	Asians	Cohort	Cerebrovascular Accident	21	NR	NR	1029	NR	NR	7
Domanski JP	2012	US	Caucasians	Cohort	Stroke	5	NR	NR	372	NR	NR	7
Ying I	2011	canada	Caucasians	Case-Control	Ischemic Stroke	103	60.4± 10.8	59/44	200	57.9 ± 11.0	97/103	7
Hamaguchi M (1)	2007	Japanese	Asians	Cohort	Ischemic Stroke	12	NR	NR	1221	NR	NR	6
Hamaguchi M (2)	2007	Japanese	Asians	Cohort	Cerebral Hemorrhage	2	NR	NR	1231	NR	NR	6

NR, not reported; ^*^Quality assessment score were assessed by the Newcastle-Ottawa Scale (NOS).

### Increased risk of CVA associated with NAFLD

**Figure 2:**
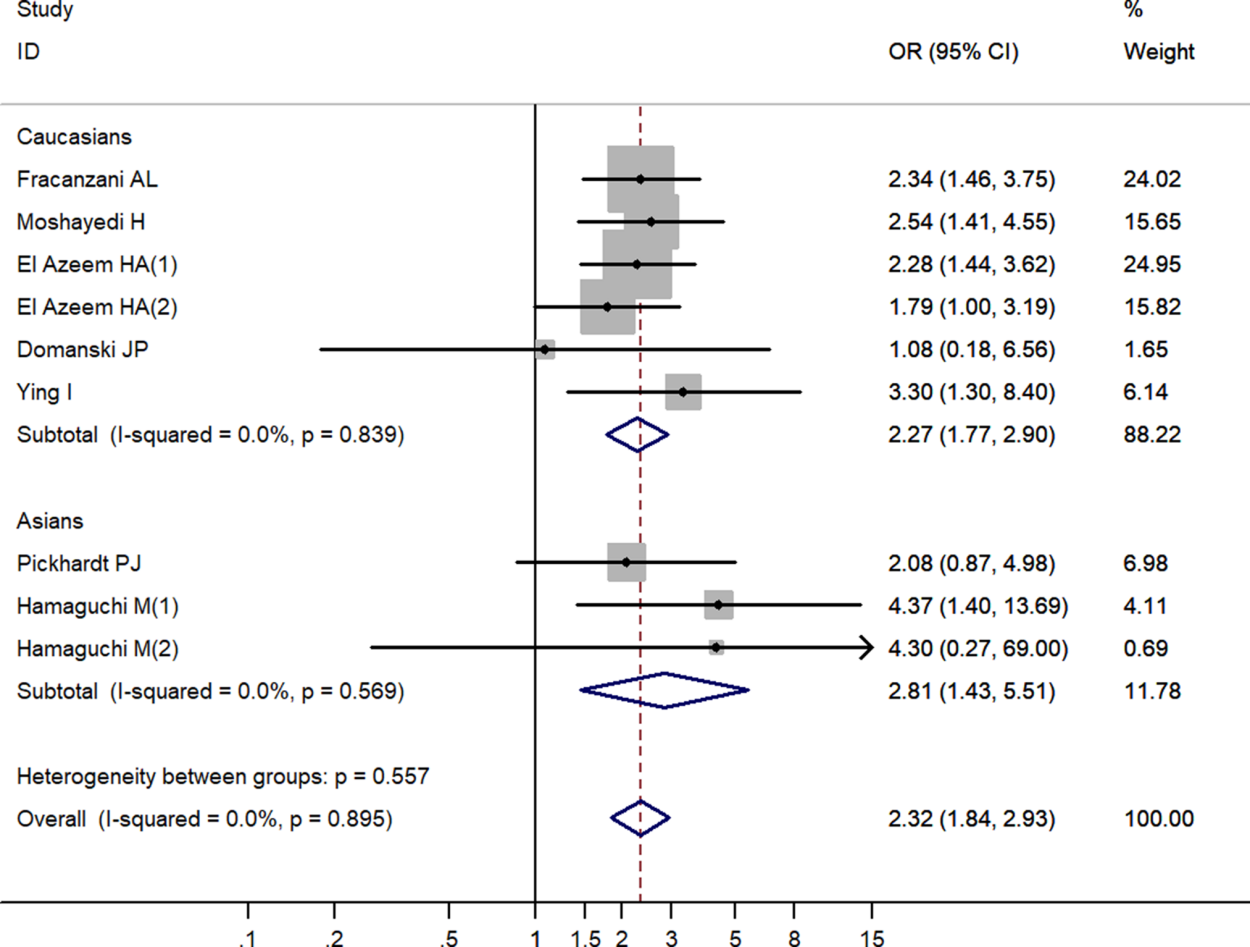
Increased risk of cerebrovascular accident related to non-alcoholic fatty liver disease by ethnicity OR, odd ratio; CI, confidence interval; P, probability.

Overall, the pooled OR suggested that NAFLD was significantly associated with elevated risk of CVA (OR = 2.32, 95% CI 1.84-2.93, *P* < 0.001) (Table 2, Figure 2). Sensitivity analysis by omitting each study did not materially modify the pooled result (data not shown).

**Table 2:** Summary meta-analysis results

Contrasts	OR(95%CI)	*P* value	Model	Heterogeneity analysis	
				*I*^2^ (%)	*P* value
**Overall**	2.32 (1.84–2.93)	< 0.001	F	0.0	0.895
**Caucasians**	2.27 (1.77–2.90)	< 0.001	F	0.0	0.839
**Asians**	2.81 (1.43–5.51)	0.003	F	0.0	0.569
**Cohort study**	2.22 (1.71–2.89)	< 0.001	F	0.0	0.833
**Case-Control study**	2.73 (1.67–4.48)	< 0.001	F	0.0	0.640
**Ischemic Stroke**	2.51 (1.92–3.28)	< 0.001	F	0.0	0.828
**Cerebral Hemorrhage**	1.85 (1.05–3.27)	0.034	F	0.0	0.544

OR, odd ratio; 95%CI, 95% confidence interval; F, fixed-effects model.

### Stratified analysis by ethnicity

We performed stratified analyses among Asians and Caucasians. Increased risk of CVA was observed related to NAFLD among both the Asian population (OR = 2.81, 95% CI 1.43–5.51, *P* = 0.003) and the Caucasian population (OR = 2.27, 95% CI 1.77–2.90, *P* < 0.001) (Table 2, Figure 2).

### Stratified analysis by study design

**Figure 3:**
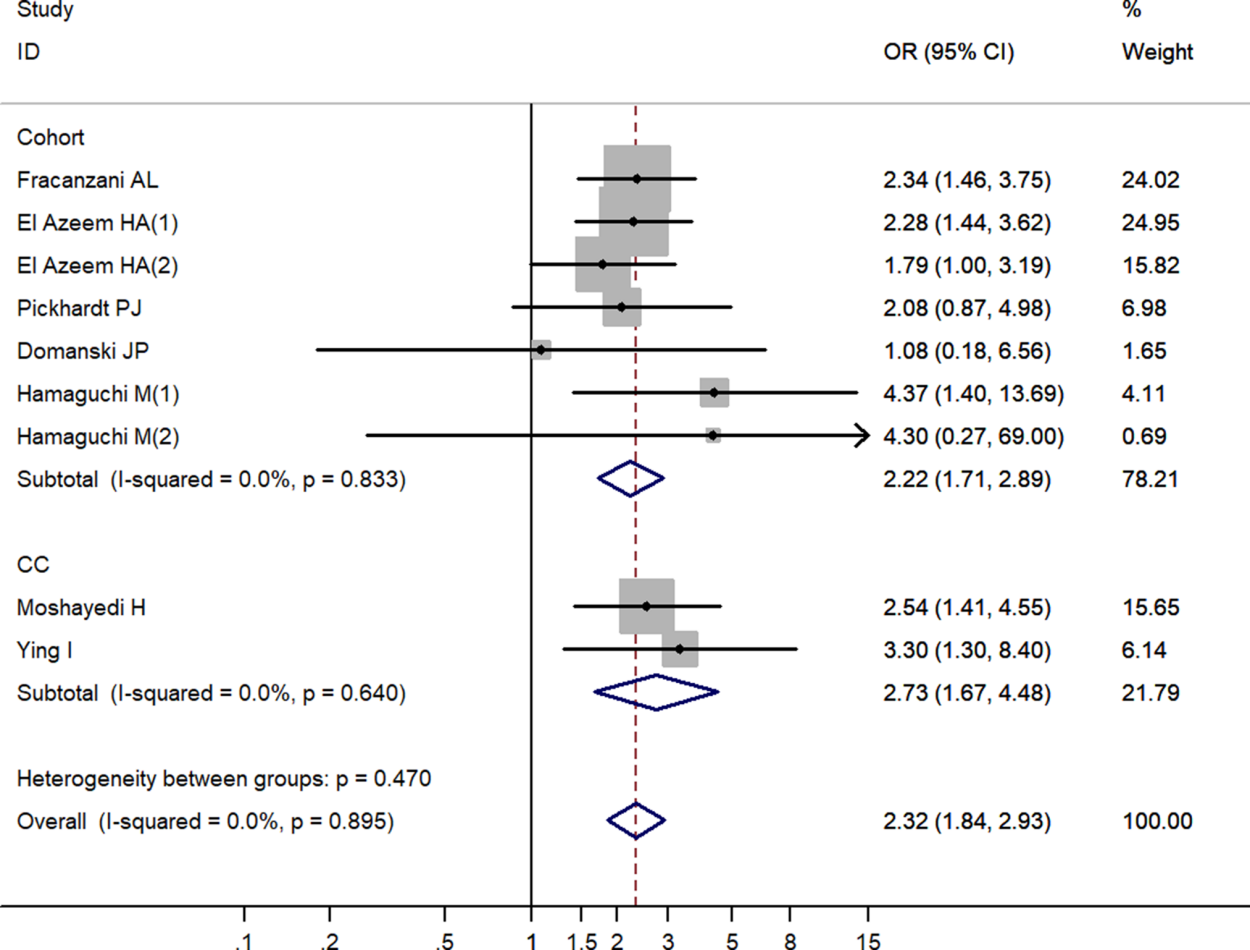
Increased risk of cerebrovascular accident related to non-alcoholic fatty liver disease by study design OR, odd ratio; CI, confidence interval; P, probability; CC, case-control study; Cohort, cohort study.

The pooled result of case-control studies showed that NAFLD could increase the risk of CVA (OR = 2.73, 95% CI 1.67–4.48, *P* < 0.001), and similar result was observed in cohort studies (OR = 2.22, 95% CI 1.71–2.89, *P* < 0.001) (Table 2, Figure 3).

### Stratified analysis by CVA classification

**Figure 4:**
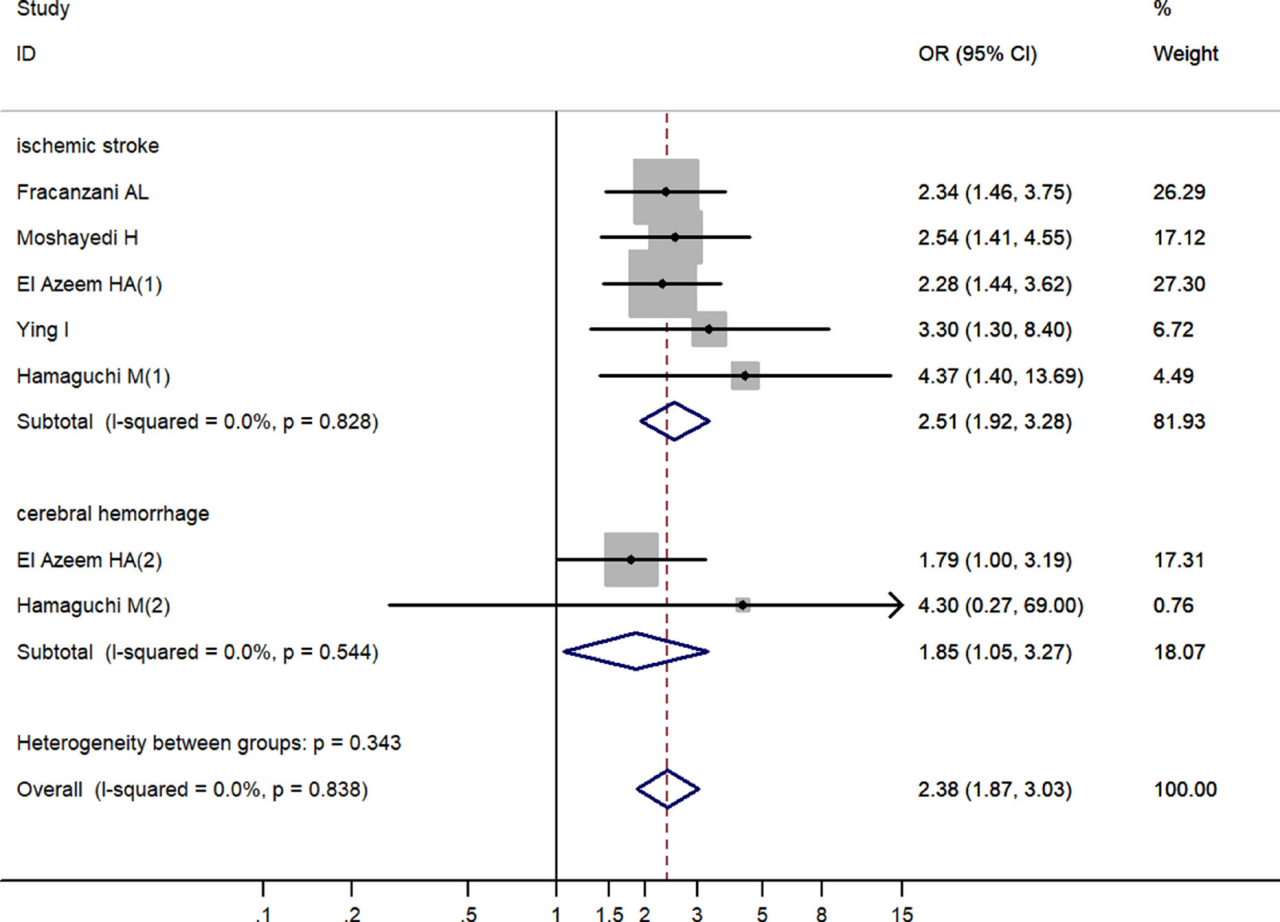
Increased risk of cerebrovascular accident related to non-alcoholic fatty liver disease by cerebrovascular accident classification OR, odd ratio; CI, confidence interval; P, probability.

Stratified analyses were conducted to estimate the modifying effect of NAFLD on different CVA, including ischemic stroke and cerebral hemorrhage. NAFLD was shown to be related to increased risk of cerebral hemorrhage (OR = 1.85, 95% CI 1.05–3.27, *P* = 0.034) and ischemic stroke (OR = 2.51, 95% CI 1.92–3.28, *P* < 0.001), respectively (Table 2, Figure 4).

### Heterogeneity analysis and publication bias risk

**Figure 5:**
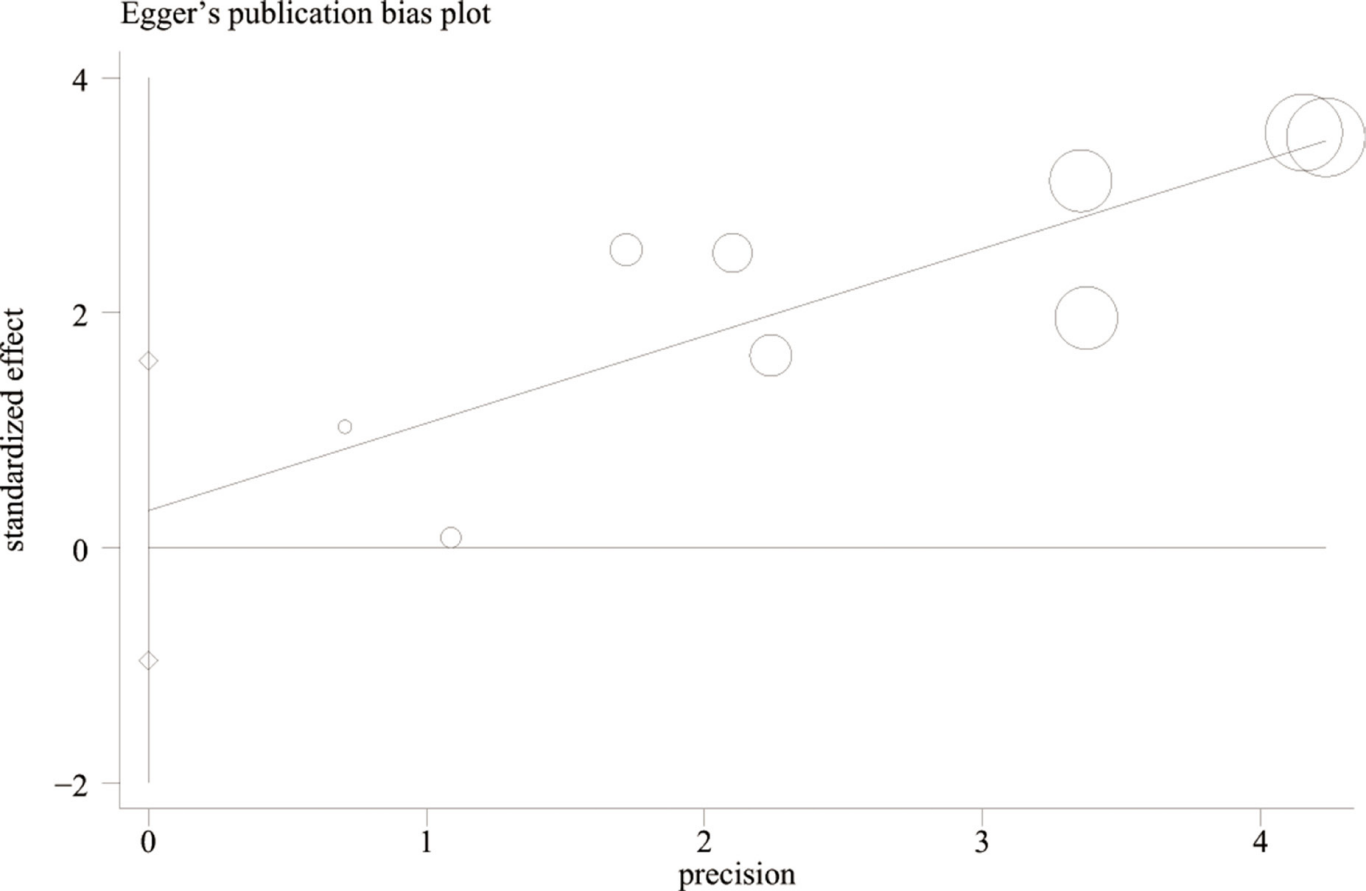
Egger's publication bias plot for the determination of publication bias risk (*P*_Egger's test_ = 0.578)

**Figure 6:**
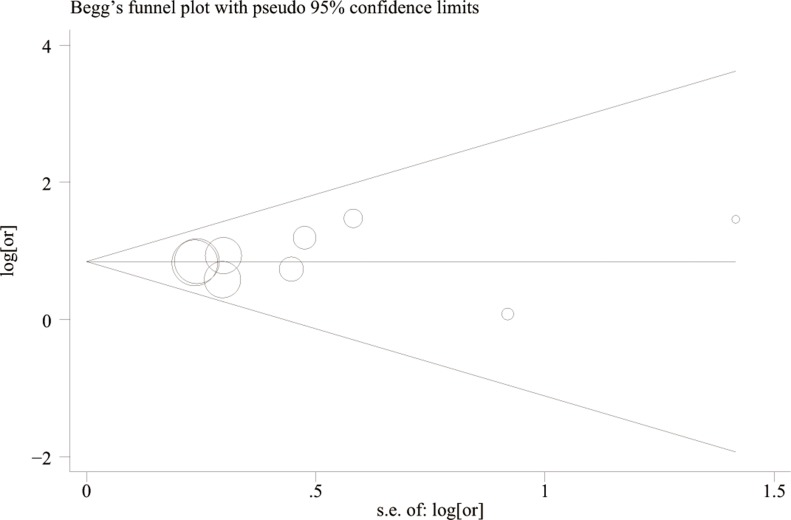
Begg's funnel plots for the determination of publication bias risk P, probability; s.e, standard error; log[or], log[odd ratio].

As shown in Table 2, we did not find obvious between-study heterogeneity between these studies. Publication bias was not detected suggested by Begg's test (*P* = 0.348) , Egger's test (*P* = 0.578) and Egger's publication bias plot(Figure 5). Visual inspection of the funnel plot suggested symmetry suggesting no publication bias (Figure 6).

## DISCUSSION

To the best of our knowledge, the effect of NAFLD on CVA development has drawn close attention for the past few years. Although some studies have investigated the association between NAFLD and CVA risk, currently available data have yielded inconsistent and inconclusive findings. Two prospective cohort studies revealed that NAFLD was a good predictor of cardiovascular disease and positively related to cardiovascular disease events, including ischemic stroke and cerebral hemorrhage, suggesting that NAFLD played a risk role in CVA [10, 11]. Reversely, Moshayedi H et al. demonstrated that NAFLD was not independently related to higher risk of ischemic stroke [5]. Pickhardt PJ and his colleagues clarified that liver steatosis was a biomarker for cardiovascular events, such as CVA, transient ischemic attack and myocardial infarction, but it was not an independent risk factor [4]. The inconsistent findings of previous studies might be attributed to diverse study design, ethnicity, clarification of CVA, statistical power, and types of diagnostic methods of NAFLD. Domanski JP found that no increased risk of cardiovascular disease was shown among the patients with NASH as compared with those with non-NASH fatty liver [12]. Taken together, the association of CVA with NAFLD remains not being clarified, probably owing to various populations and diverse study design. Meta-analysis could shed some light on the contradictory findings across independent studies. Therefore, we carry out this meta-analysis and perform stratified analyses by ethnicity, study design and CVA classification, respectively. The pooled results suggest that NAFLD significantly confers risk effect on CVA, and the effect of NAFLD on the susceptibility to CVA was further confirmed in the stratified analyses by race, study design and CVA classification. We provide strong evidence, for the first time, for the risk role of NAFLD in CVA.

Individuals with NAFLD, characterized by liver inflammation, are at higher risk to develop liver fibrosis and cancer [13]. Recently, the influence of inflammation in stroke has received sustaining attention, and it was proved that inflammatory mechanisms play a key role in the pathogenesis and progression of atherosclerosis and stroke [14]. Increased inflammatory cytokines have been observed in the patients with NAFLD. The activation of pro-inflammatory signaling pathways in NAFLD may lead to liver chronic inflammation [15]. A systemic inflammatory status with activation of proinflammatory transcription factors and infiltration of considerable proinflammatory cytokines plays a decisive role in the development of atherosclerosis and CVA [16, 17]. The activation and upregulation of NF-κB resulting from hepatic steatosis will increase the expression of several proinflammatory mediators and amplify the systemic inflammation [18, 19]. The sustained inflammatory status may promote arteriosclerosis and plaque in the arterial wall and the development of CVA. In our study, the pooled ORs suggested that NAFLD was a risk factor for CVA. However, we failed to the effect of common inflammatory mediators or transcription factors on the association between NAFLD and CVA risk due to unavailable data in previous publications.

A previous study identified that Asians and Hispanics showed a higher degree of steatosis than Whites and other ethnicities [20]. The prevalence and histological features of NAFLD also have obvious ethnic difference [21, 22]. It was well documented that the association between NAFLD and enhanced inflammation only remained significant in white people but not Chinese, African American and Hispanic people after multivariable adjustment [23]. Taken together, ethnicity may be a significant confounding factor when estimating the relationship of NAFLD with CVA risk. El Azeem HA indicates that NAFLD played a risk role in CVA [11], while Moshayedi H demonstrated that NAFLD is not related independently to ischemic stroke [5]. Hamaguchi M proved that NAFLD was positively related to cardiovascular disease events [10], while Pickhardt PJ clarified that liver steatosis was not an independent risk factor [4]. We performed the stratified analysis by ethnicity to determine the risk of CVA related to NAFLD among different ethnic populations. The pooled results showed that increased risk of CVA was observed related to NAFLD among both Caucasian and Asian populations. Some other confounding factors may also confer modifying effects on the association between NAFLD and CVA risk, such as study design and CVA classification. Nonetheless, stratified analysis by study design in our study showed that NAFLD could increase the risk of CVA in both case-control studies and cohort studies. However, more relevant studies are warranted for further investigation.

Ischemic stroke and cerebral hemorrhage are the main forms of CVA. We conducted the stratified analysis by CVA classification to investigate the potential role of NAFLD in different CVA mainly including ischemic stroke and cerebral hemorrhage. We found that NAFLD was related to increased risk of both ischemic stroke and cerebral hemorrhage, suggesting similar pathogenic molecular mechanisms for ischemic stroke and cerebral hemorrhage. It has been well established that hypertension is a major risk factor for developing CVA and also predisposes to the development of atherosclerosis. CVA, including cerebral infarction, cerebral hemorrhage and lacunar infarction, are the severe complications of atherosclerosis [24, 25].

Some limitations must be considered in our meta-analysis. Firstly, as mentioned above, studies regarding the cerebral hemorrhage risk among the Asian population were not sufficient for a precise estimate due to relatively small sample size. Secondly, the adjusted factors in every study were different, and bias might be introduced in the present study. Last but not the least, CVA is a multifactorial disease with complex pathogenic factors, including genetic polymorphisms, environmental exposures, history of the family, and so on. A combining effect between NAFLD and other common risk factors may be critical in the development of CVA, which warrants further elucidation.

Overall, the results showed that NAFLD was significantly associated with elevated risk of CVA. In the stratified analyses by ethnicity, study design and CVA classification, we also observed the similar results. Thus, our study, firstly, suggests that NAFLD is a significant risk factor for CVA among both Asians and Caucasians. However, more relevant studies are warranted for further investigation in the future.

## MATERIALS AND METHODS

### Search strategy

We followed the Guidelines for Meta-Analyses of Observational Studies in Epidemiology (MOOSE) group to identify eligible studies. A comprehensive literature search was performed to identify acceptable studies on the association of NAFLD and CVA risk in PubMed, Embase, Cochrane Library databases, Wanfang and CNKI databases from their inception up to Sep 30, 2017. The following terms were used: “cerebrovascular accident” or “cerebrovascular disease” or “stroke” or “ischemic stroke” or “cerebral hemorrhage” or “cerebral infarction” or “brain attack” or “paralytic”; and “fatty liver disease” or “steatohepatitis” or “hepatic steatosis”. In addition, the reference lists of all retrieved studies and the PubMed link “related articles” were also screened for additional studies.

### Inclusion criteria

The inclusion criteria were as follows: (1) observational studies, including case-control study and cohort study; (2) providing enough information for odds ratio (OR) or relative risk (RR) or hazard ratio (HR) and 95% confidence intervals (95% CI) or raw data. Duplicate publications, reviews, case reports, irrelevant study or study with overlapping data were all excluded. The qualities of all included studies were assessed using the Newcastle-Ottawa Scale (NOS). Studies were graded as good quality if they awarded 6 to 9 stars; fair if they awarded 3 to 5 stars; and poor if they awarded less than 3 stars.

### Data extraction

Two researchers carried out the literature search independently by using a standardized data extraction form, and the disagreements were resolved by discussion.

The data were as follows: first author, year of publication, country, ethnicity, study design, number of cases and controls, matching factors, adjusted factors, RR or HR or OR with 95% CI, disease classification.

### Statistical analysis

The strength of the relationship between NAFLD and CVA was estimated by calculating the pooled ORs with 95% CIs. ORs and 95% CIs were calculated with SPSS19.0 by use of the raw data in the single study. Cochrane's Q statistics and *I*^2^ statistic tests were used to evaluate heterogeneity of the OR across studies [26, 27]. The random-effects model was applied when the between-study heterogeneity was significant [28]; otherwise, the fixed-effects model was used when the between-study heterogeneity was not significant [29]. We conducted stratified analyses by study design, ethnicity and disease classification for further investigation. Sensitivity analysis was also carried out by sequential omission each study. Begg's funnel plot and Egger's test were adopted to assess the publication bias in the present meta-analysis [30, 31]. Statistical analysis was performed by using STATA 12.0 software (StataCorp, College Station, TX, USA). All *P* values were two-sided, and *P* < 0.05 suggested statistical significance.
